# XY and ZW: Is Meiotic Sex Chromosome Inactivation the Rule in Evolution?

**DOI:** 10.1371/journal.pgen.1000493

**Published:** 2009-05-22

**Authors:** Satoshi H. Namekawa, Jeannie T. Lee

**Affiliations:** 1 Howard Hughes Medical Institute, Department of Molecular Biology, Massachusetts General Hospital, Boston, Massachusetts, United States of America; 2 Department of Genetics, Harvard Medical School, Boston, Massachusetts, United States of America; Fred Hutchinson Cancer Research Center, United States of America

**Figure 1:**
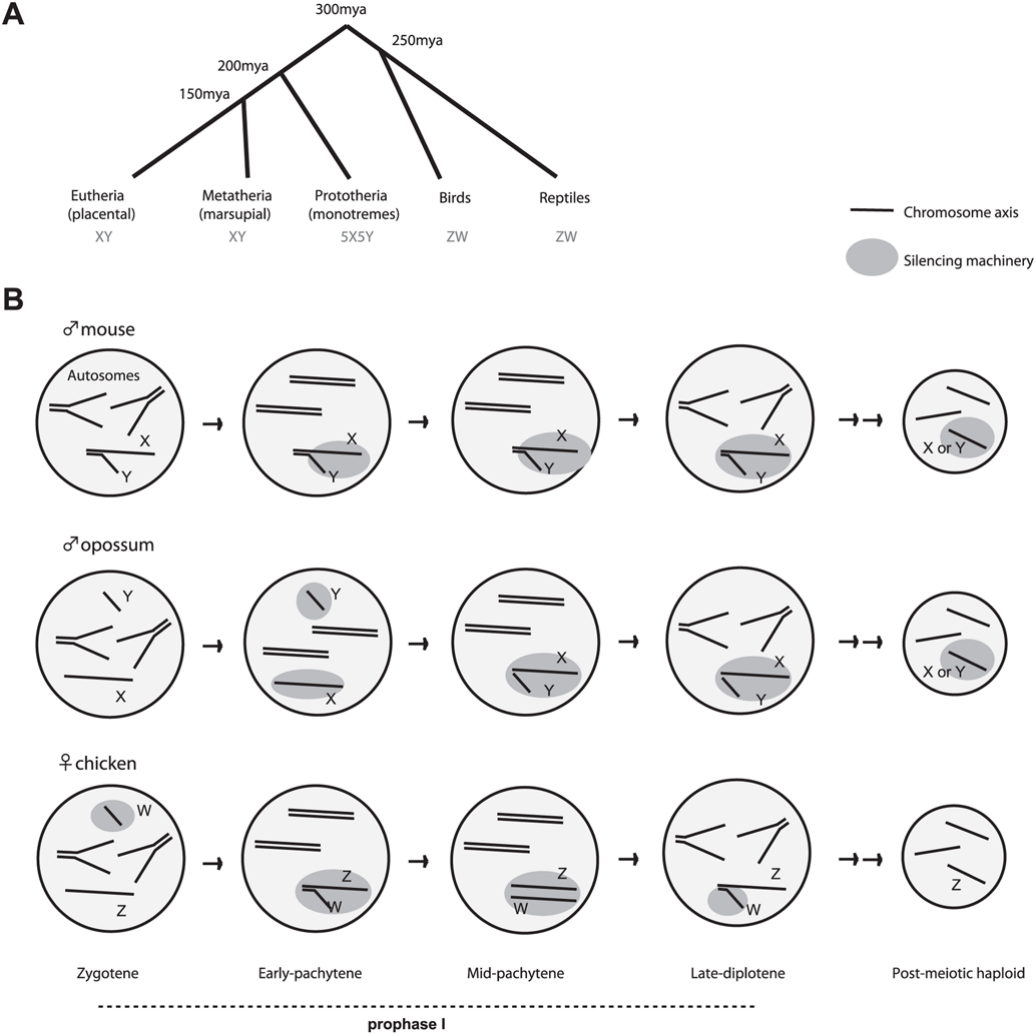
MSCI in the bird and the mammal. (A) Divergence of birds from mammals approximately 300 million years ago coincides with the split between XY and ZW sex determination schemes. (B) Schematic representation of MSCI in birds, marsupial mammals, and placental mammals. Unlike the X and Y, all of the Z and W are co-localized and held together by a proteinaceous bond during mid-pachytene (intertwined), although there is no homology and no genetic exchange takes place. ZW inactivation is also limited to prophase I, whereas XY inactivation persists into the post-meiotic period.

The sex chromosomes are among the most rapidly evolving and most diverse genetic systems in all of biology. Students of model organisms may, however, have the false impression that there is only one chromosomal mechanism of specifying sex. Among the best-studied metazoans, the XY system is indeed the rule, with inheritance of two X's determining the female sex (XX), and inheritance of an X and a Y specifying the male sex (XY) [1]. In this system, females produce only one type of oocyte (X), whereas males produce two types of sperm (X and Y). However, sex is not always determined this way. Throughout evolution, the XY system has co-existed alongside the lesser known ZW system, a scheme exemplified by members of the avian clade who diverged from Mammalia 300 million years ago (Figure 1) [2],[3]. In birds, females are the heterogametic sex, as females have one Z and one W chromosome (ZW) and can therefore produce two types of gametes (Z or W oocytes). By contrast, males are ZZ and can produce only one type of gamete (homogametic)—the Z-bearing sperm. In XY and ZW systems, the homologous sex chromosomes are genetically unequal due to suppression of homologous recombination and accumulation of deleterious mutations on one chromosome of the heterogametic sex [1]. In the XY system, it is the Y that genetically degenerates; in the ZW system, it is the W. Today, the mammalian X carries over three times more genes than the Y does, whereas the chicken Z carries over ten times more than the W.

There are two intriguing consequences of having unequal sex chromosomes. The first relates to dosage imbalance or X- or Z-borne genes between males and females. A need to correct for this imbalance has led to co-evolution of “dosage compensation” in many organisms that use the XY system, such as mammals, fruit flies, and worms [4],[5]. In mammals, dosage compensation involves transcriptional inactivation of one X chromosome in the female. The second consequence of unequal sex chromosomes is the absence of a full pairing partner during meiosis in the heterogametic sex [6]. During meiosis, homologous chromosomes pair (align), synapse (held by the synaptonemal complex), and exchange genetic material via homologous recombination. But for sex chromosomes, pairing occurs either partially or not at all. The X and Y of eutherian mammals pair through their pseudoautosomal regions, but the X and Y of marsupial mammals lack significant homology and come together without synapsis [7],[8]. Lack of pairing triggers meiotic silencing of unsynapsed chromatin (or unpaired DNA) (MSUC or MSUD) [9]–[11], which is an ancient genome defense mechanism that silences sequences without pairing partners [12]. Mammalian MSUC/MSUD results in meiotic sex chromosome inactivation (MSCI), by which the X and Y alone become transcriptionally inactivated during the first meiotic prophase [6], [13]–[15]. MSCI is not confined to mammals, as metazoans as diverse as the fruit fly [16], grasshopper [17], and the nematode worm [18] also demonstrate MSCI (grasshopper and worm males are XO, with the Y having completely degenerated).

How universal are dosage compensation and MSCI? Analyses in chickens have reached the consensus that Z genes may only be partially equalized between ZZ and ZW individuals, although the mechanism of dosage compensation remains unclear [2],[3]. Until now, no evidence of MSCI had been found in birds [19],[20]. In this issue of *PLoS Genetics*, Schoenmakers et al. have re-examined bird oogenesis and found that MSCI actually occurs in chickens [21]. This discovery has a number of implications for the evolution and developmental behavior of sex chromosomes.

Chicken MSCI is both similar and different from MSCI in XY animals [21]. Like mammal and worm MSCI, chicken MSCI occurs during the first meiotic prophase (divided into leptotene, zygotene, pachytene, and diplotene) and is marked by chromatin changes. Schoenmakers and colleagues observed heterochromatic marks and exclusion of Pol-II on both Z and W and verified, by quantitative reverse-transcriptase (RT)-PCR analysis of a handful of Z and W genes, that the genes are expressed at lower levels during pachytene than in zygotene and diplotene, as is observed for murine X and Y genes [13],[14]. Although how much of Z and W is silenced remains to be investigated, these similarities imply a conserved mechanism based in part on MSUC/MSUD.

There are intriguing differences as well, one of which is in the timing relative to chromosome synapsis. In eutherian mammals, MSCI coincides with the failure of synapsis during pachytene [9],[10]. On the other hand, chicken MSCI precedes synapsis of Z and W. Thus, chicken MSCI may be based as much on “unpairing” as on “asynapsis.” Interestingly, this feature of ZW MSCI is similar to opossum MSCI, which occurs in early pachytene before X and Y colocalization [7]. Therefore, in chickens and opossum, a homology search mechanism—rather than asynapsis itself—might be the trigger for MSCI.

MSCI in birds and mammals also differs in terms of what chromatin changes occur. In the mouse and the opossum, chromatin changes and transcriptional silencing take place concurrently throughout nonhomologous regions of both sex chromosomes. Microarray analysis has shown that very few genes escape MSCI in mice. Both X and Y exclude Pol-II and are coated by heterochromatic marks such as H3-K9me3 and HP1, as well as by MSUC-associated marks such as γH2AX. By contrast, chicken W-inactivation slightly precedes Z-inactivation, leading Schoenmaker et al. to hypothesize that MSCI occurs by the spreading of heterochromatin from W to Z [21]. (An alternative is that W and Z inactivation occur independently, which cannot be excluded.) Additionally, γH2AX is absent on the ZW pair during pachytene and accumulates only after separation of ZW during late pachytene—and only on the Z. Thus, ZW inactivation seems different from XY inactivation in the eutherian (mouse), but may partially resemble XY inactivation in marsupial (opossum), the mammalian clade that is evolutionarily closer to birds (Figure 1). These differences suggest that MSCI in birds and mammals both borrowed from MSUD/MSUC, but independent evolutionary origins led to unique innovations as well.

A final major difference is found in XY and ZW transcriptional fates after meiosis. In mice, the effects of MSCI are felt long after meiosis is finished—i.e., the X and Y remain heterochromatic and transcriptionally suppressed during the entire post-meiotic period [14],[22],[23]. In two other XY (or XO) species examined to date (grasshopper and worm), post-meiotic silencing is also observed [17],[18]. By contrast, bird MSCI is very transient and is lost by late diplotene [21]. Thus, post-meiotic silencing is not an absolute consequence of MSCI. One school of thought argues that persistence of silencing is an active process that evolved for a specific purpose in mammals [7],[14].

Why do the X and Y remain suppressed after meiosis? And why does MSCI occur in the first place? Given that early spermatogenesis genes are enriched on the mammalian X and that several spermiogenesis genes can also be found there [24], transcriptional suppression is especially puzzling. One thought suggests that MSCI was driven by sexual antagonism, i.e., a male germline response to a “feminized” X that might adversely affect spermatogenesis [19]. Another possibility is that MSCI is merely an evolutionary relic of MSUD/MSUC; persistence into the post-meiotic period might occur by default. The fact that ZW inactivation is transitory, however, argues against the latter idea. It is also possible that MSCI evolved to suppress recombination between the nonhomologous sex chromosomes. Silent post-meiotic sex chromatin could then have been exploited and extended to deal with dosage compensation in the early XX embryo of marsupial and eutherian mammals, both of which display imprinted paternal X-chromosome silencing [7],[14],[15]. Post-meiotic silencing is remarkably heritable in the worm, and possibly also in the grasshopper as well [17],[18]. The inheritance of a pre-inactivated X from the paternal germline would be an effective way of achieving dosage balance between XX and XY offspring. The absence of post-meiotic silencing in chickens is consistent with this hypothesis, as dosage compensation is not robust in avians studied to date [2],[3].

Birds are mammals' closest relatives on the evolutionary tree (Figure 1). Around 300 million years ago, avian and mammalian genetics evolved such that the ZW system was used in the avian progenitor and the opposite XY system was used in the mammalian progenitor. While the XY system has been intensively investigated, many questions remain regarding the ZW system. How is sex determined by the Z and W, and why did MSCI evolve in birds if its effects are as ephemeral as they seem? Does MSCI bear any relationship to dosage compensation? The cumulative evidence suggests that MSCI is universal among organisms bearing heteromorphic sex chromosomes. Regardless, the ZW system will be a useful model, because avian MSCI is so transient, and it should facilitate teasing out the *raison d'etre* of MSCI, separated from post-meiotic silencing and its after-effects.
